# Personality Traits in Fibromyalgia (FM): Does FM Personality Exists? A Systematic Review

**DOI:** 10.2174/1745017901814010223

**Published:** 2018-09-28

**Authors:** Ciro Conversano, Laura Marchi, Rebecca Ciacchini, Claudia Carmassi, Bastianina Contena, Laura Maria Bazzichi, Angelo Gemignani

**Affiliations:** 1 Department of Surgical, Medical and Molecular Pathology, Critical and Care Medicine, University of Pisa, Pisa, Italy; 2 Department of Clinical and Experimental Medicine, Rheumatologic Clinic, University of Pisa, *via* Roma 67, 56100 Pisa, Italy; 3 Department of Clinical and Experimental Medicine, University of Pisa, *via* Roma 67, 56100 Pisa, Italy; 4 Department of Health Science, University of Florence, Florence, Italy

**Keywords:** Fibromyalgia, Personality traits, Personality model, Personality, Chronic pain, FM patients

## Abstract

**Introduction:**

Fibromyalgia (FM) is the second most common rheumatic disease with many effects on patient's quality of life. It has been described as a chronic condition characterized by widespread musculo-skeletal pain, sleep disorders and prominent fatigue. Regarding the role of personality factors in fibromyalgia, researchers have focused both on personality traits and psychopathological aspects showing inconsistent results. In particular, several studies have examined the role of alexithymia in FM patients, a trait of personality characterized by difficulty in identification, recognition and description of emotions and feelings, while others have focused on a specific type of personality, such as type D personality (distressed personality). Other studies investigated personality in FM patients referring to Cloninger’s model, a psychobiological model of personality that includes both temperamental and character dimensions of personality. Analyzing scientific literature on this subject seems well suited to provide a critical review of the latest studies and their results.

**Methods:**

The method used for this review satisfies the Preferred Reporting Items for Systematic Review and Meta-Analyses (PRISMA). We identified PsycInfo and PubMed as databases for our research.

**Results:**

Personality is studied under many aspects and a reference model is not always present. Many studies underline high levels of alexithymia and type D personality in FM patients but when depression is controlled, these results do not differ from those of healthy controls.

**Conclusion:**

Studies that use a comprehensive model of personality present a different theoretical approach and use alternatively the Big-Five model, Eysenck’s and Cloninger’s models. The use of a comprehensive model of personality and the control of psychopathological disorders, such as anxiety and depression, seem to be very relevant for a better understanding of a specific personality profile associated with fibromyalgia.

## 1 INTRODUCTION

Fibromyalgia has been described as a chronic condition characterized by widespread musculoskeletal pain, sleep disorders and prominent fatigue [1]. The prevalence of fibromyalgia as a rheumatic disease has shown to be between 0.2 and 6.6% in the general population, between 2.4 and 6.8% in women and between 0.7 and 11.4% in urban areas [2]. Worldwide, researchers are still debating on the as yet unclear etiopathogenesis of fibromyalgia which could comprise genetic predisposition, environmental factors, an influence on the neuroendocrine functions and the contribution of immune system [3]. A recent work [4] revealed how certain gene polymorphisms (especially those influencing serotoninergic, dopaminergic and catecholaminergic systems) could be directly involved in fibromyalgia development, with an influence on susceptibility and symptoms severity. Jiao and colleagues [5] observed the role of physical trauma and infection as a precipitating factor, while another study [6] suggested that regulation of pain process can be influenced by viral or bacterial infection throughout the activation of a specific type of cytokines. In his critical review, Di Tella M, *et al.* [7] highlighted that central sensitization could play an important role in fibromyalgia syndrome; in fact, the high sensitization to noxious stimuli can lead to hyperalgesia or allodynia and equate different clinical conditions (central sensitivity syndromes). In a recent review [8] fibromyalgia has been described as a central sensitization syndrome characterized by chronic widespread pain. There are other factors that could influence both the onset and the course of fibromyalgia: Eating habits, psychic stress response (as a psychological variable) and lack of social relationships [9]. Nevertheless, patients suffering from this condition have to deal with heterogeneous symptom manifestations, non-resolving treatments and a high prevalence of comorbidity suggesting a complicated diagnostic process [9]. Fibromyalgia often occurs in comorbidity with psychiatric conditions of various kinds, including mood disorders and anxiety disorders [10-13]. Specifically, some studies highlighted high percentages of major depression (comorbidity rates range from 20 to 80%), dysthymia and Generalized Anxiety Disorder [14-16] in FM patients.

### 1.1 Criteria and Assessment

Specialists have used the well-known 1990 criteria of the American College of Rheumatology [17] for approximately 20 years to diagnose fibromyalgia [18]. These criteria required a minimum of three months of extended widespread pain (with no distinctions regarding side of body) and a sensitivity to digital palpation on at least 11 particular body sites named tender points. Many studies have criticized the choice of tender points, suggesting that it is not possible to detect pressure pain in these points while they represent a good measure for prolonged negative effects of stress. A recent study [19] aimed to explain why the so-called tender points pressure method has no diagnostic sensitivity. This approach seems to not be capable of equally representing men and women's physical condition due to their demonstrated differences concerning tender point location and quantity in the body. The new criteria were revealed in 2010 and the main purposes were to simplify the diagnostic process and to include non-painful symptoms, such as sleep disturbances (especially non-restorative sleep) and various cognitive disorders (such as the so-called ‘fibro fog’). Another purpose was to shift the overview of fibromyalgia from a peripheral condition concept to a systemic one, to support the multifactorial and complex nature of the disease. For this, new criteria include self-reported evaluations of a Widespread Pain Index and a Symptom Severity Scale which also comprehend cognitive functioning and sleep quality assessment [20]. The new female: male ratio diagnosed by using the new criteria is 2:1 [21] and the assessment is made by a global evaluation using a shorter symptoms checklist among other things.

### 1.2 Personality Profile

As a matter of fact, many researchers have studied the role of personality factors in fibromyalgia. Many studies have focused both on personality traits or particular characteristics and psychopathology aspects in a narrow sense. In this regard, a recent meta-analysis [22] highlighted significant statistical differences in clinical profiles between FM patients and healthy controls. More specifically, the research group identified three different sub-groups in FM patients: One group with the neurotic triad profile (elevation on hypochondrias, depression and hysteria scores), another group with a normal profile (absence of significant high-ranked scales) and a group with a psychopathological profile (high scores in at least 4 scales). Another recent analysis [22] investigating several different studies on prevalence of borderline personality disorder in patients with FM and chronic fatigue, showed inconsistent results on prevalence estimation (from 1% to 16.7%). Kayhan *et al.* [23] demonstrated that, with the exception of histrionic personality disorder, there are no significant differences between FM group and controls. Likewise, researches dealing with personality traits in fibromyalgia patients have at times shown inconsistent results. Several studies have focused on alexithymia, a personality trait characterized by difficulty in identifying and describing emotions and feelings in FM patients [24] while others have analyzed a specific type of personality, such as type D personality (distressed personality). Previously exposed personality traits included high negative affectivity (tendency to experience negative feelings) and social inhibition (low propensity to express emotions to others, owing to social disapproval or rejection [25].

Many studies that centered on aspects such as neuroticism and/or extroversion [26, 27] highlighted that only some patients have shown significant differences in profiles when compared with healthy controls. Furthermore, various researchers discussed the specific role played by personality traits on fibromyalgia: Some studies investigated personality as a predisposing factor in the onset and maintenance of the disease [28, 29] while others as a moderation factor compared to the disease’s adaptation process. For this reason, analyzing scientific literature on this subject seems to be fundamental in order to provide a critical review of the latest studies and their results.

## 2 MATERIALS AND METHODS

The method used for this review satisfies the Preferred Reporting Items for Systematic Review and Meta-Analyses (PRISMA) [30]. We identified PsycInfo and PubMed as databases for our research and selected *fibromyalgia* and *personality* as specific keywords, combined with the Boolean operator *and*. Specific inclusion criteria were as follows: a) the article was written in English and b) published in peer review journals, c) from 2010 to 2017 (the time span was selected based on the recent changing of international diagnostic criteria for fibromyalgia). Initially, the research returned a total number of 366 articles: From this opening corpus we deleted duplicated articles and corrigendum (n=12). At this stage some specific keywords were used to refine the results, in particular the Boolean operator *not*, to exclude articles about:

- *adolescents* (n=37),
- *treatment* and *treatment* *outcome* and *pharmacological* *treatment* (n=99)
- *psychometric* *properties* of evaluation instruments or *questionnaires* (n=124)
- *etiologic* aspects of rheumatic diseases and *single* *case* *studies* (n = 29)
- other *rheumatic* *diseases* and *comorbidities* (n=41).

The final number of articles enrolled in this study was 24, of which the full texts were examined independently. We decided to exclude a review about borderline personality disorder [31] in fibromyalgia because of its focus on a specific psychopathological disorder, a meta-analysis about the use of Minnesota Multiphasic Personality Inventory (MMPI) [22] in patients with fibromyalgia for its focus on a specific instrument and, lastly, a letter to the editor about perfectionism [32]. The articles examined for this systematic review were definitively 21. (Table **1**). Fig. (**1**) presents a flow chart depicting the literature search process.

### 2.1 Analysis Criteria

The authors decided on these analysis criteria:

- definition of fibromyalgia and diagnostic criteria used for the study;
- aspects of personality analysed in terms of definition, instruments and hypotheses.

## 3 RESULTS

- Regarding Definition and Diagnostic Criteria of Fibromyalgia Twenty studies [2, 3, 5, 6, 19-26, 28, 29, 33-41] defined fibromyalgia as a syndrome characterized by chronic widespread pain and sensitivity to digital palpation and only one study referred to fibromyalgia as a rheumatic disease [35]. Nineteen of these studies [2, 3, 5, 6, 19, 23, 25, 26, 28, 29, 33-41] add to this basic definition the presence of fatigue, sleep disorders, cognitive impairment and emotional distress. Furthermore, 5 studies [5, 19, 29, 35, 36] highlighted the presence of sensitivity to tender points (11/18) as described by ACR criteria for FM diagnosis in 1990 [21] while only two studies referred explicitly to the new ACR criteria [13] for its definition and diagnosis. From a methodological perspective, all studies enrolled participants who had been diagnosed with FM: In particular, 7 studies [5, 19, 24, 26, 33, 34, 41] recruited participants among those who had been diagnosed by expert rheumatologists and fibromyalgia hospital unit outpatients. In three studies [35, 36, 39], participants were selected from previously used databases. Ten studies clearly used the ACR criteria to select participants with FM; in one study the participants were described as people who meet the ACR criteria, without other specifications [37], 7 referring to ACR criteria of 1990 [2, 3, 6, 23, 25, 29, 39] and only 3 used the 2010 criteria [28, 38, 30].
- Regarding Aspects of Personality The examined studies focused on various aspects of personality associated with FM and highlighted different roles for these aspects compared to FM. Five articles used the term “personality traits” to identify specific psychopathological elements in FM patients, pointing out the role of personality disorders, anxiety, depression as characteristics of a subgroup of patients [35-37, 40, 41]. Other studies examined some specific personality traits such as alexithymia, type D personality, emotional regulation, neuropsychological aspects and dysfunctional beliefs, and used a comprehensive model of personality such as that of Eysenck, Cloninger or the big-five model.

As far as neuropsychological aspects are concerned, three studies of the present review focused on this aspect [19, 26, 29]. The reasoning of the authors concerned the complex relationship between the comprehension and regulation of emotional states and the neurological networks that operate the ability required for social cognition. From the authors’ point of view, this executive function related to planning, working memory, attention and inhibition could possibly explain the hypersensitivity to pain and the difficulties in emotional recognition and regulation. Di Tella *et al.* [34], starting from the model of Miyake *et al.* [42], explained the subcomponents of Executive Function (EF) evaluating the social-cognitive profile of patients with FM and the relationship between the performance on cognitive tasks with that on social tasks. Results showed a significant impairment in social cognition skills in people with FM who highlighted at the same time a substantial independence of this impairment from executive function deficits. Miró *et al.* [43] explored the relationship between attention functioning and the main symptoms of FM with differences between genders and with healthy controls in three attention networks (alertness, orienting and executive control). Findings revealed a general impairment in attention networks for these patients, similar in both males and females. Rosselló *et al.* [44] evaluated the autonomic and central nervous response during affective startle modulation using the motivational priming hypothesis. They found out that patients are characterized by relevant impairment in affective modulation in response to affective state induction and they suggested the presence of alterations in both attentional and emotional aspects of information processing that could explain the main symptoms of FM.

## 3.1 Alexithymia

Five studies examined alexithymia in FM patients [5-7, 17, 20]. Alexithymia has been described as a personality trait that includes difficulty in identification and description of emotions and feelings, together with an externally oriented cognitive thinking style [45]. The Toronto Alexithymia Scale (TAS-20), a self-report 20-item questionnaire, was chosen as the fundamental measure of alexithymia in all of these studies [45].

Di Tella *et al.* [35] examined the relationship between alexithymia and pain (both sensory and the affective component of pain) in 159 patients with fibromyalgia. The study showed that alexithymic patients exhibited higher level of pain intensity and pain experience (both sensory and affective dimensions) when compared to non-alexithymic ones (controls). Moreover, alexithymic patients showed higher levels of anxiety, depression and emotional distress compared with controls: When controlling anxiety, depression and emotional distress, no correlation was found. Emotional distress was the only predictor on intensity of pain and neither anxiety/depression or alexithymia showed a significant role in pain intensity.

The study of Montoro and del Paso [27] revealed that FM patients show higher levels of alexithymia compared with the healthy control group. Similarly, in Martinez *et al.* [41] alexithymia was shown to play an important role in predicting the affective aspects of pain. The study found FM group more alexithymia than in controls and when anxiety, depression and pain appraisal variables were controlled, alexithymia lost meaningfulness as a predictor of pain experience and sleep quality. On the other hand, anxiety demonstrated to be highly qualified in predicting pain experience, as depression was the best choice for sleep quality. Difficulty in identifying emotions has proved to have a moderate role in the relationship between pain catastrophizing levels and anxiety. Di Tella and colleagues [24] showed higher levels of alexithymia in FM sample compared with controls; moreover, a positive correlation between alexithymia, anxiety, depression and pain was found.

Ablin, *et al*. [28] showed two different personality profiles in a fibromyalgic sample: The first was characterized by higher well-being, more adaptive coping strategies and lower levels of alexithymia with a similar prevalence of type D personality when compared with the general population; the second qualified with lower levels of well-being, less adaptive coping strategies, higher levels of alexithymia and more prevalence of type D personality.

Moreover, the author assessed personality aspects from a psychobiological point of view. Van Middendorp *et al.* [46] found that on this peculiar type 56.5% of FM patients showed distressed personality while type D was associated with mental and physical health.

Specifically, the negative affective component was strongly related to the mental health of the sample and type D personality appeared highly prevalent in this group. In their contribution, Ablin and colleagues [28] studied the role of alexithymia and type D personality in fibromyalgia and chronic fatigue syndrome, and found in some patients maladaptive profiles with high level of alexithymia and high frequency of type D personality. Additionally, the authors examined all the patients’ personality profiles using a specific model that also other studies adopted: The psychobiological model [28, 38, 39, 47].

## 3.2 Models of Personality

As with the study of Ablin *et al.* [28] two other studies investigated personality in FM patients referring to Cloninger’s model, a psychobiological model of personality, that includes both temperamental (harm avoidance, novelty seeking, persistence and reward dependence) and character dimensions of personality (self-transcendence, self-directedness and cooperativeness [39, 47]. Temperament and Character Inventory (TCI) was applied in all these studies. Ablin *et al.* [28] showed a maladaptive profile of some patients characterized by a high level of harm avoidance and a low level of cooperativeness, self-directedness, persistence and reward dependence. Santos *et al.* [47] observed a higher level of harm avoidance and self-transcendence and lower levels of novelty seeking, self-directedness, reward dependence and cooperativeness in patients with FM when compared with controls. When anxiety and depression were controlled, only novelty seeking was associated with FM. Personality is investigated also using the Big-Five model and the model of Eysenck. Three studies [2-4, 12] examined FM patients’ personality according to the five-factor model which includes 5 main dimensions of personality: Extroversion/introversion, openness to experience/closed-minded, conscientiousness/impulsivity, neuroticism/emotional stability, agreeableness/antagonism.

Two studies [26, 29] employed the Big Five Personality inventory and only one [43] the Neo-Personality Inventory (NEO-P-R). Bucourt *et al.* [26] demonstrated how FM patients had higher scores in neuroticism, openness and agreeableness when compared with other rheumatic diseases. Furthermore, significant correlations between neuroticism and pain, and impulsivity and pain in FM group were found. Malin and Littlejohn [29] proposed a top-down model that assumes several psychological variables (mastery, neuroticism, anxiety) as predictors of stress which could influence FM symptoms (pain, fatigue and sleep disturbances); moreover, both anxiety and neuroticism were associated with stress. In the study of Da Silva *et al.* [33] various items were selected to represent peculiar FM patients’ personality characteristics in comparison with controls; for the most part, different experts sorted items in the field of neuroticism, revealing good consistency.

Montoro and del Paso [27] assessed the three personality dimensions (neuroticism, psychoticism and extroversion) according to Eysenck’s personality model in FM patients with the Eysenck Personality Questionnaire Revised-Abbreviated (EPQR-A). The FM group showed higher levels of neuroticism and no significant difference on psychoticism and extroversion levels. Finally, two studies focused on specific aspects associated with personality psychopathologic traits. The first study [48] compared the personality core-beliefs in patients with FM and major depressive disorder, showing an absence of specificity on these characteristics. The second one [23] instead analysed the role that personality disorders may have in predicting the quality of life of people with rheumatic diseases, showing that it could affect both quality of life perception and coping strategies.

## 4 DISCUSSION AND CONCLUSION

Personality traits are often studied in patients with rheumatic diseases and, most of all, in those with fibromyalgia. However, the literature on this topic presents inconsistent results. The proposed systematic review underlines some specific characteristics of the recent literature and highlights some relevant key-points for future researches. Firstly, there were many differences in the definitions of fibromyalgia and also in patient recruitment in the studies. Some authors only enrolled participants who were referenced in diagnosis, while some others used patients with a diagnosis formulated with ACR criteria of 1990 [17] and yet others with ACR criteria of 2010 [21].

Secondly, personality is studied under many aspects and a reference model is not always present. Many studies underline high levels of alexithymia and type D personality in FM patients but when depression is controlled, these results do not differ from those of healthy controls. Studies that use a comprehensive model of personality present a different theoretical approach and use alternatively the Big-Five model, Eysenck’s and Cloninger’s models. Obviously, when the theoretical model of personality changes, also the measures change, and that could explain the difference found between results. Therefore, the contrasting results are probably due to the use of different explicative models and diverse measures of personality. Malin and Little [40] observed that the scientific research examining personality characteristic in FM patients is limited by the differences in instrument design as well as by the limited data on clinical aspects of FM patients. They also did not find any study showing a specific personality profile in FM patients. In the study of Lundberg *et al.* [48] both aspects, such as temperament and character, of Cloninger’s TCI test were found different in FM personality.

In some cases, personality is studied in terms of psychopathology, with a completely different approach: Measures and results show how personality disorders can influence subthreshold dysfunctional traits. In these cases, personality is conceptualized as a feature, affecting patients’ quality of life and their response to treatment in general. Although in many studies FM patients are compared with healthy controls and personality differences are found, when comparison is made with other disorders with controlled depression, personality traits appear to be less relevant than before.

From this analysis, for future research, the relevance of a clear definition of fibromyalgia is evinced, together with the necessity to enrol participants on the basis of more recent criteria. Moreover, the use of a comprehensive model of personality and of psychopathological disorders, such as anxiety and depression, seems to be very relevant for a better understanding of a specific personality profile associated with fibromyalgia, also because of the high prevalence of Axis I psychopathologies in FM patients [45].

Obviously, some limits are present in our study: Our choice of scientific databases, such as PsycInfo and PubMed and the inclusion criteria (such as the English language), could exclude important studies in the field. Furthermore, most of the studies include female participants and thus no gender differences are controlled. For future research, it might be useful to extend the analysis of the literature on this topic, focusing on gender differences in personality aspects and their relationship with fibromyalgia.

## Figures and Tables

**Fig. (1):**
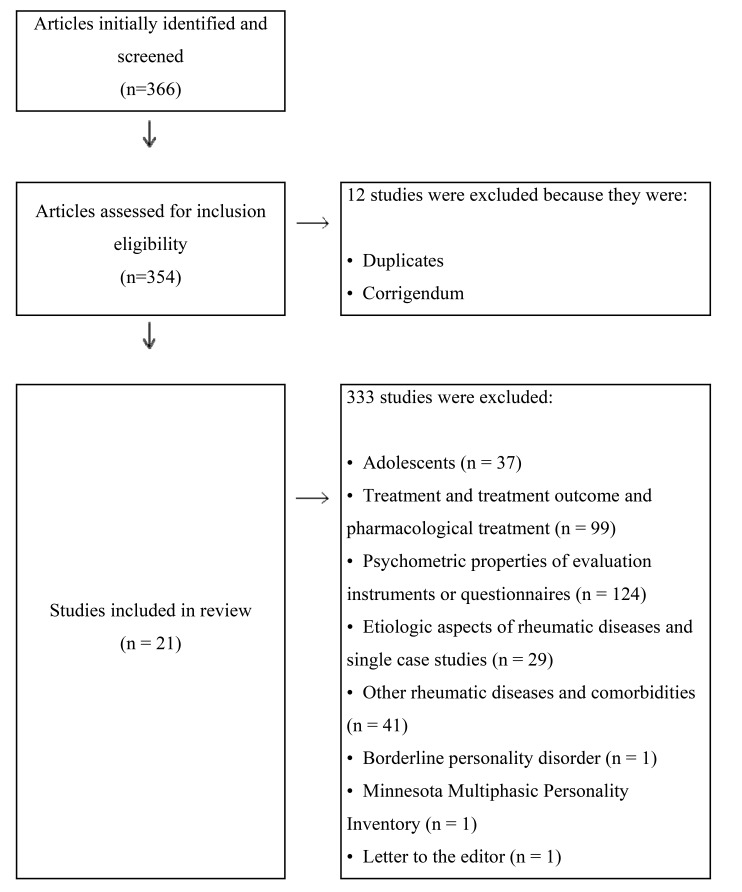
Flow chart for literature search and screening results.

**Table 1:** Studies examined in this systematic review.

Authors	Title	Year		No.	Model	
Ablin, *et al.*	Distinctive personality profiles of fibromyalgia and chronic fatiguesyndrome patients.	2016		1	Psychobiological model	
Bucourt, **et al.**	Comparison of the Big Five personality traits in fibromyalgia and otherrheumatic diseases.	2016		2	Big Five	
Da Silva, **et al.**	Can health care providers recognise a fibromyalgia personality?	2017		3	Big Five	
Di Tella, *et al.*	Theory of mind and emotional functioning in fibromyalgia syndrome: An investigation of the relationship between social cognition and executive function.	2015		4	Big Five	
Di Tella, *et al.*	Coping strategies and perceived social support in fibromyalgia syndrome: Relationship with alexithymia.	2017		5	Alexithymia	
Di Tella, *et al.*	Pain experience in fibromyalgia syndrome: The role of alexithymia andpsychological distress.	2017		6	Alexithymia	
Garcia-Fo ntanals, *et al.*	Vulnerability to psychopathology and dimensions of personality in patients with fibromyalgia.		2017	7	Alexithymia	
Gonzales, *et al.*	Fibromyalgia characterization in apsychosocial approach.		2014	8	Psychobiological model	
Gumà-Uri el, *et al.*	Impact of IPDE-SQ personality disorders on the healthcare and societal costs of fibromyalgia patients: A cross-sectional study.		2016	9	Psychopathology	
Kayhan, *et al.*	Sexual dysfunction, mood, anxiety, and personality disorders in female patients with fibromyalgia.		2016	10	Psychopathology	
Leombruni, *et al.*	Harm avoidance and self-directedness characterize fibromyalgic patients and the symptom severity.		2016	11	Psychobiological model	
Malin & Little john	Psychological factors mediate key symptoms of fibromyalgia through their influence on stress.		2016	12	Big Five	
Martinez, *et al.*	Relationships between physical symptoms, emotional distress, and pain appraisal in fibromyalgia: The moderator effect of alexithymia.		2015	13	Alexithymia	
Miro, *et al.*	Men and women with fibromyalgia: Relation between attentional function and clinical symptoms.		2014	14	Neurospychological aspects	
Montoro & Reyes del Paso	Personality and fibromyalgia: Relationships with clinical, emotional, and functional variables.		2015	15	Neuroticism	
Montoro, *et al.*	Alexithymia in fibromyalgia syndrome.		2016	16	Alexithymia	
Rossello, *et al.*	Affective modulation of brain and autonomic responses in patients with fibromyalgia.		2015	17	Neuropsychological aspects	
Santos, *et al.*	The influence of depression on personality traits in patients with fibromyalgia: A case-control study.		2016	18	Psychobiological model	
Taymur, *et al.*	Personality-related core beliefs in patients diagnosed with fibromyalgia plus depression: A comparison with depressed and healthy control groups.		2014	19	Dysfunctional beliefs	
Uguz, *et al.*	Quality of life in rheumatic patients: The impact of personality disorders.		2015	20	Psychopathology	
